# Influence of differences in bone morphology on the distribution patterns of subchondral bone density across the trapeziometacarpal joint

**DOI:** 10.1038/s41598-022-16746-7

**Published:** 2022-07-20

**Authors:** Yukinori Tsukuda, Yuichiro Matsui, Kaori Endo, Yuki Matsui, Daisuke Kawamura, Norimasa Iwasaki

**Affiliations:** 1 Department of Orthopaedic Surgery, Otaru General Hospital, Wakamatsu 1-1-1, Otaru, Hokkaido 047-8550 Japan; 2 Faculty of Dental Medicine, Hokkaido University, Kita 13 jo Nishi 7 chome, Kita-ku, Sapporo, Hokkaido 060-8586 Japan; 3 Department of Orthopaedic Surgery, Faculty of Medicine and Graduate School of Medicine, Hokkaido University, Kita 15 jo Nishi 7 chome, Kita-ku, Sapporo, Hokkaido 060-8638 Japan

**Keywords:** Bone, Cartilage

## Abstract

We aimed to clarify the effects of morphological patterns of the trapezium and first metacarpal on the distribution of subchondral bone density across the articular surface of the trapeziometacarpal (TMC) joint using computed tomography osteoabsorptiometry. Thirty-three patients with normal TMC joints were evaluated. The percentages of the high-density areas in the radial-dorsal and ulnar-volar regions of the trapezium were significantly higher than that in the ulnar-dorsal region, and that of the ulnar-dorsal region of the first metacarpal was significantly lower than in the other three regions. The percentage of the high-density area of the radial-dorsal region of the trapezium and trapezial inclination (TI) showed a significant positive correlation, and the percentages of the high-density areas in the ulnar-dorsal and ulnar-volar regions had significant negative correlations with TI at the articular surface of the first metacarpal. These results indicate that bony morphologic differences in the trapezium affect the distribution pattern of subchondral bone density through the TMC joint.

## Introduction

The trapeziometacarpal (TMC) joint plays a key role in thumb movement, and is characterized by an incongruent biconcave saddle joint. This joint allows not only abduction–adduction and flexion–extension movements, but also circumduction, allowing opposition using the thumb and other fingers^1^. Osteoarthritis of the TMC joint is a common disease that can lead to debilitating conditions for the hand. The prevalence of radiographic TMC osteoarthritis is around 90% for adults ≥ 80 years old^2^. TMC osteoarthritis causes problems such as thumb pain or limitations to range of motion, so a number of anatomical and biomechanical studies have attempted to clarify the pathomechanisms underlying osteoarthritis of this joint^3–5^. Pelligrini et al. analyzed degenerative patterns of the TMC joint at the time of TMC joint arthroplasty, and suggested that arthritic changes were related to the translation of the metacarpal on the trapezium, mainly because of laxity of the beak ligament^6^. However, these issues remain poorly understood.

The TMC joint performs diverse motions and plays an important role in pinching with first metacarpal pronation. Eaton et al. suggested that compressive loading leads to chondromalacia of the articular cartilage in the radial-dorsal region of the thumb basal joint^7^. Ateshian et al. suggested that the ulnar-volar and radial-dorsal regions of the trapezium were the most common sites of thin cartilage, using stereophotogrammetry of mainly the lateral pinch position^8^. Loading areas of the TMC joint are thus known to change via various thumb movements. In a cadaveric study by Koff et al., cartilage wear was found to occur in areas with greater contact stress, and TMC osteoarthritis tended to arise in these overloaded areas^9^. Schneider et al. showed that peak stress differed between men and women, as peak stress locations are more variable in women during grasp and jar twist than in men^10^. However, much remains unclear about contact stress on the TMC joint under physiological loading because of difficulties in the direct measurement of force distributions in the joints of living organisms. Actually, the TMC joint bears complex external forces in activities of daily living. Understanding the biomechanical effects under physiological loading conditions is thus important for clinicians. These complex biomechanical conditions make it difficult to analyze the stress distributions through the TMC joint in cadaveric studies.

Although assessing TMC joint stress under actual loading conditions remains challenging, we attempted to assess the distributions of subchondral bone density using computed tomography osteoabsorptiometry (CT-OAM). A few studies have shown that stress distributions under physiological loading conditions through a joint are indirectly assessed with the distributions of subchondral bone density that can be measured using CT-OAM^11,12^. Previous studies have demonstrated that CT-OAM can potentially be used to evaluate cumulative stress distribution patterns, measuring the distribution patterns of subchondral bone density, indirectly in various joints^13–19^. We believe that the analysis of subchondral bone density has the potential to be a useful tool to study alterations in stress distribution through the TMC joint under load.

Several reports have examined correlations between the bony morphology of the TMC joint and TMC osteoarthritis. Bettinger et al. suggested that advanced TMC osteoarthritis was associated with increased trapezial tilt, representing radial inclination of the trapezium at the TMC joint. Trapezial tilt angles without arthritis and with severe arthritis (stage III and IV of the Eaton classification) were 42° and 50°, respectively^20^. Miura et al. reported that volar inclination of the first metacarpal at the TMC joint was greater in a TMC osteoarthritis patient group, and that increased volar inclination of the first metacarpal at the TMC joint in patients with TMC osteoarthritis was associated with a predisposition to dorsal translation^21^. They concluded that the morphology of the first metacarpal correlated with TMC osteoarthritis, although those studies were radiographic. Whether morphological differences of the trapezium and first metacarpal influenced the pathogenesis of TMC osteoarthritis under physiological loading conditions has remained unclear. We hypothesized that morphological differences in the trapezium and first metacarpal would be factors affecting the distribution patterns of subchondral bone density through the TMC joint. The aims of this study were to assess the distribution patterns of subchondral bone density across the TMC joint and to evaluate the relationships between morphological patterns of the trapezium and first metacarpal and the distribution of subchondral bone density across the articular surface.

## Methods

### Patients

CT image data of the TMC joints of 33 patients with various diseases not affecting the TMC joint were collected for analysis from June 2018 to September 2019. Demographic data are shown in Table 1. Exclusion criteria were as follows: pre-existing fracture of the trapezium or first metacarpal; radiographic findings indicating osteoarthritis of the TMC joint; history of any hand surgery; or history of systemic disease such as inflammatory arthritis.

**Table 1:** Characteristics of study group participants.

	Demographic data (SD)
Total no	33
Sex	
Male	11
Female	22
Age (years)	68.2 (12.1)
Disease	
Distal radius fracture	23
Distal radioulnar joint osteoarthritis	3
Lunate dislocation	1
Ulnar abutment syndrome	1
Distal radius malunion	1
Proximal phalangeal fracture of the index finger	1
Proximal phalangeal fracture of the little finger	1
Carpal tunnel syndrome	1
Wrist bruise	1
Trapezial inclination (°)	8.1 (2.7)
Volar tilt (°)	9.5 (3.6)

### Definition of bony morphology of the trapezium and first metacarpal

**Figure 1:**
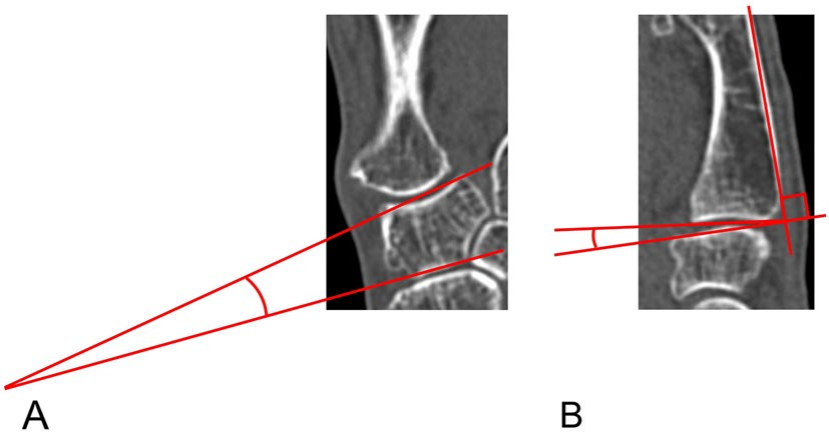
Computed tomography. (**A**) Trapezial inclination (TI) on the coronal view. (**B**) volar tilt (VT) on the sagittal view.

The angle between lines connecting the two edges of the articular surface of the trapezium in the TMC and scaphotrapezial joints was defined as trapezial inclination (TI) on the coronal view of the first metacarpal (Fig. 1)^22^. The angle between the tangent to the dorsal cortex of the first metacarpal and the line connecting the two edges of the articular surface of the first metacarpal of the TMC joint was defined as volar tilt (VT) on the sagittal view (Fig. 1)^22^.

### Computed tomography osteoabsorptiometry

**Figure 2:**
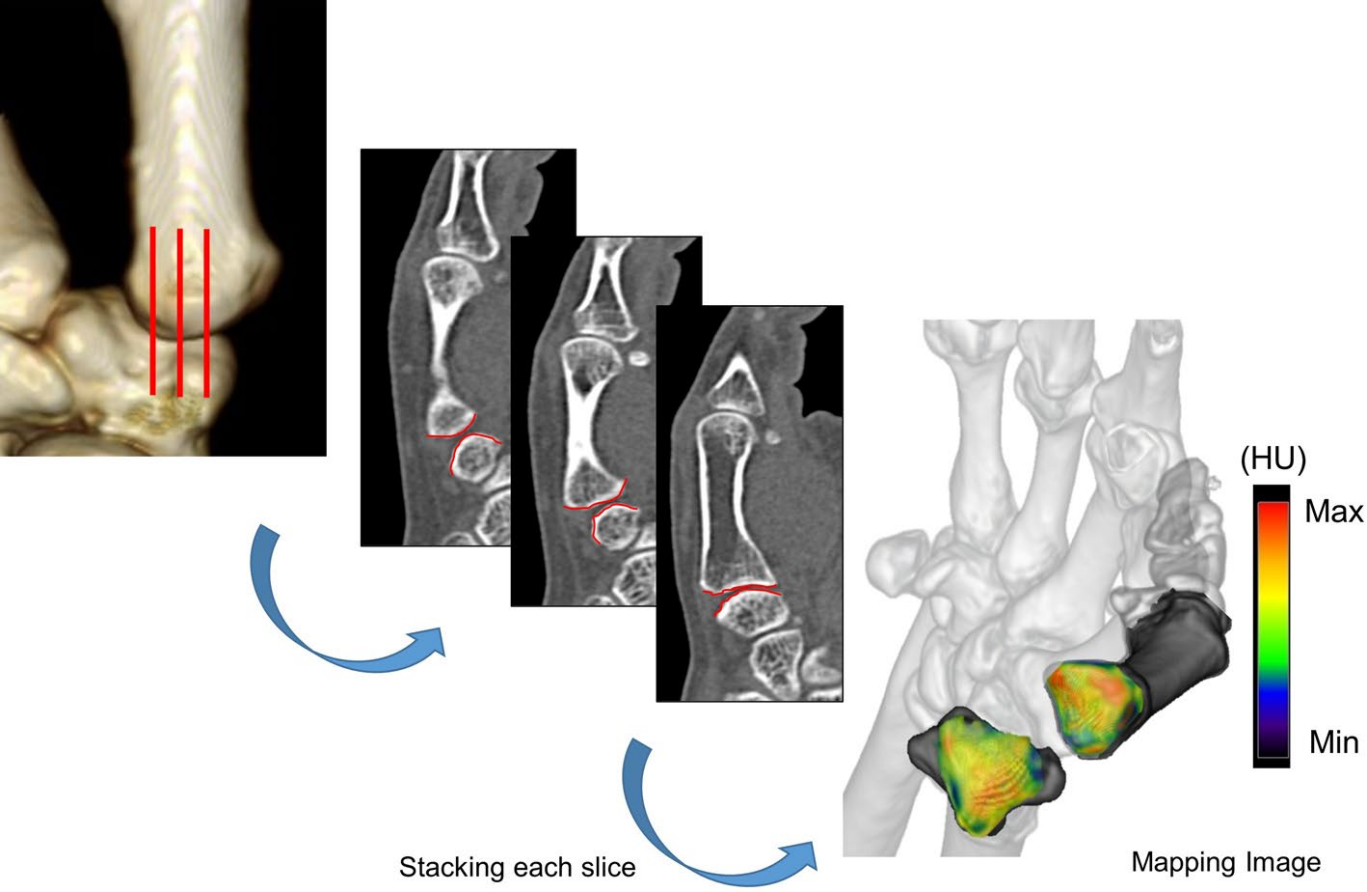
Identification of subchondral bone regions for articular surfaces of the trapezium and first metacarpal using customized software. In each sagittal slice, X-ray attenuation (in Hounsfield units) is measured in subchondral bone at each coordinate point within a 1-mm interval, and the distribution pattern in the subchondral bone is represented as a surface-mapping image.

A high-resolution (512 × 512 pixel matrix) helical CT scanner (Multi Detector CT; Canon Inc., Tokyo, Japan) was used to obtain sagittal images of the trapezium and first metacarpal. CT imaging data were transferred to the Aquilion One image analysis system (Toshiba Medical Systems, Otawara, Japan) for further evaluation. A 3-dimensional bone model was created from the axial image stack. Sagittal views at intervals of 1 mm were acquired from the multiplanar reconstruction model. Customized software was used for further evaluation. The region of interest in sagittal images was selected to include the entire subchondral bone layer of the articular surface of the trapezium and first metacarpal. The maximum increment point in Hounsfield units (HU; where water is 0 HU and compact bone is 1000 HU) from the joint surface was selected as the starting point of the region of interest, and the maximum point in HU was selected automatically from the starting point to a depth of 2.5 mm. This maximum point (1 pixel = 0.5 mm) was defined as subchondral bone. We determined the radiodensity of the identified subchondral bone region at each coordinate point at 1 mm intervals. Measured density at each coordinate point was mapped with a color scale, in which red indicates the greatest bone density and deep blue indicates the lowest bone density. Measurement and mapping were repeated in each slice. Finally, a 2-dimensional mapping image that projected the distribution of subchondral bone density was obtained (Fig. 2).

**Figure 3:**
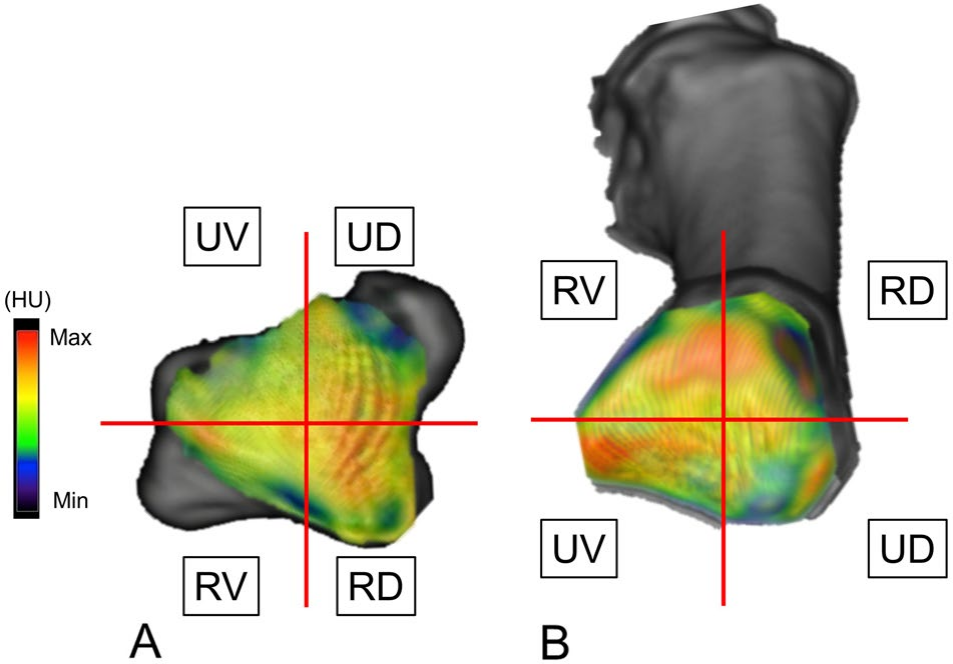
The image shows regions using quantitative analysis of bone density mapping data in the TMC joint of the right hand. (**A**) the articular surface of the trapezium. (**B**) the articular surface of the first metacarpal. *RD* radial-dorsal, *RV* radial-volar, *UD* ulnar-dorsal, *UV* ulnar-volar.

Quantitative analysis of mapping data focused on the locations of high-density areas in the articular surfaces of the trapezium and first metacarpal. The high-density area was defined as the region containing the coordinate points representing the upper one-third of Hounsfield unit values out of the total area of the subchondral bone, as in some previous reports^14,15,17^. We drew a line parallel to the sagittal plane at the middle point from the most radial point to the most ulnar edge and another line parallel to the coronal plane from the most dorsal point to the volar edge. We divided the image into four regions using these lines. Articular surfaces of the trapezium and first metacarpal were divided into four regions: radial-dorsal (RD), radial-volar (RV), ulnar-dorsal (UD), and ulnar-volar (UV) (Fig. 3). The percentage of the high-density area occupying the total area was calculated. Moreover, percentage of the high-density areas of the four regions at the trapezium and first metacarpal were also calculated. The obtained data were compared among RD, RV, UD and UV regions in each bone. Correlations between high-density areas of each region and TI or VT were investigated in each bone.

Before the analysis, intraobserver consistency in measurement of subchondral bone density was assessed by calculating the coefficient of variation, as the standard deviation divided by the mean value, expressed as a percentage. When we measured the subchondral bone density of the entire articular surface of the trapezium and first metacarpal, one observer (Y. T.) performed five measurements to calculate the coefficient of variation. However, only the first measurements were used in the analysis of this study. The coefficient of variation of the percentage of the high-density area among the results of five measurements was calculated.

### Statistical analysis

Results are shown as mean and standard deviation (SD). One-way analysis of variance was used to test the significance of differences in mean values for continuous variables. Correlations were analyzed using Pearson’s product-moment correlation between the high-density area of each region and TI or VT. Differences were considered significant for values of *P* < 0.05. All statistical analyses were performed using JMP Pro version 10.0 statistical software (SAS Institute, Cary, NC, USA, available at https://www.jmp.com/ja_jp/software.html).

### Ethical approval

Ethical approval for this retrospective study was provided by the institutional review board of Otaru General Hospital. Written informed consent was obtained from all patients for publication of this report and accompanying images. The methods were carried out in accordance with the principles of the Declaration of Helsinki.

## Results

Coefficients of variation for measurement of the percentage of the high-density area in articular surfaces of the trapezium and first metacarpal calculated by the observer were 9.1% and 6.6%, respectively. These values were less than 10%, which is considered acceptable for comparison study purposes^18,23^. Therefore, all measurements in this study were performed by a single observer.

Percentage of the high-density areas are shown in Table 2. Percentage of the high-density areas of the RD and UV regions of the trapezium were significantly higher than that of the UD region in the articular surface (UD, 24% (SD 18%) vs. RD, 35% (SD 14%), *P* = 0.02; vs. UV, 37% (SD 17%), *P* < 0.01), and that at the UD in the articular surface of the first metacarpal was significantly lower than those of the other three regions (UD, 25% (SD 18%) vs. RD, 38% (SD 19%), *P* = 0.04; vs. RV, 43% (SD 18%), *P* < 0.01; vs. UV, 38% (SD 19%), *P* = 0.03).

**Table 2:** Percentage of the high-density areas in each region at articular surfaces of the trapezium and first metacarpal.

	Total	RD	RV	UD	UV	*P*-value
**Trapezium**						
%, mean (SD)	34 (12)	35* (14)	28 (14)	24 (18)	37* (17)	< 0.01
**First metacarpal**						
%, mean (SD)	37 (13)	38* (19)	43* (18)	25 (18)	38* (19)	< 0.01

*RD* radial-dorsal region, *RV* radial-volar region, *UD* ulnar-dorsal region, *UV* ulnar-volar region.

*P < 0.05 versus UD.

Table 3 shows correlation coefficients between TI or VT and the high-density area. The percentage of the high-density area of the RD region and TI showed a significant positive correlation in the articular surface of the trapezium (*P* < 0.01), and percentages of the high-density areas of the UD and UV regions displayed significant negative correlations with TI in the articular surface of the first metacarpal (UD, *P* = 0.03; UV, *P* < 0.01). No significant correlations were seen between percentage of the high-density area and VT in both articular surfaces.

**Table 3:** Correlation coefficients between percentage of the high-density area and trapezial inclination or volar tilt.

	Correlation coefficient Trapezium	*P*-value	Correlation coefficient First metacarpal	*P*-value
**TI**				
All	0.233	0.19	− 0.307	0.08
RD	0.517	< 0.01*	− 0.058	0.75
RV	0.310	0.08	0.047	0.79
UD	0.145	0.42	− 0.383	0.03*
UV	0.030	0.88	− 0.453	< 0.01*
**VT**				
All	0.109	0.54	− 0.108	0.55
RD	0.124	0.49	− 0.251	0.15
RV	0.306	0.08	0.093	0.60
UD	− 0.220	0.22	− 0.273	0.12
UV	0.084	0.64	0.119	0.50

*TI* trapezial inclination, *VT* volar tilt.

*P < 0.05.

## Discussion

A few biomechanical studies relevant to the TMC joint have been performed^4,24–26^, although much remains unclear about contact stress on the TMC joint under physiological loading because of difficulties in direct measurement of force distribution in the joints of living organisms. In this study, percentage of the high-density areas of the RD and UV regions in the articular surface of the trapezium were significantly higher than that of the UD region. The percentage of the high-density area of the UD region in the articular surface of the first metacarpal was significantly lower than those of the other three regions. From a previous report, incongruent joint contact caused by lateral pinch in the presence of metacarpal pronation led to localized stress peaks in the UV and RD regions of the trapezium^8^. Cartilage wear was not predisposed to occur in the UD region of the first metacarpal, even if TMC osteoarthritis progressed^9^. Furthermore, if TMC osteoarthritis progresses, degeneration develops in the RD and UV regions of the articular surface of the trapezium^9^. Our results support these previous reports. The percentage of the high-density area of the RD region and TI showed a significant positive correlation in the articular surface of the trapezium, and those of the UD and UV had significant negative correlations with TI in the articular surface of the first metacarpal, although no significant correlations were found between percentage of the high-density area and VT in both articular surfaces. These results indicate that the bony morphology of the trapezium may affect the distributions of subchondral bone density of the TMC joint surface, and an articular surface of the trapezium with higher inclination toward the radial side would gather stress on the radial-dorsal side. Only one previous report has described the distributions of subchondral bone density of the TMC joint measured by CT-OAM, which is relevant to pressure distributions on the first metacarpal caused by Bennett’s fracture^27^, although no previous studies have examined the normal TMC joint. We believe that this study is the first to show a correlation between the distributions of subchondral bone density of the normal TMC joint and trapezium morphology.

**Figure 4:**
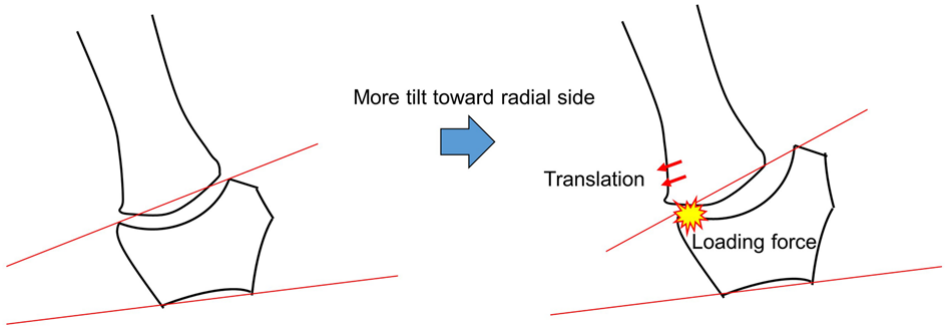
Mechanism of the distribution of subchondral bone density in the articular surface of the trapezium. The distribution of subchondral bone density is more likely to gather in the radial-dorsal region because of radial translation of the first metacarpal, if trapezial inclination is increased.

Esplugas et al. showed that various ligaments around the TMC joint prevented translation of the first metacarpal toward the radial side. In particular, dorsal ligaments such as the posterior oblique, dorsoradial and dorsal central ligaments became engaged in preventing the first metacarpal from translating radial-dorsally^24^. Because the TMC joint has a structure prone to translation of the first metacarpal radial-dorsally in the absence of the stability provided by these ligaments, the first metacarpals of individuals having higher TI may be more likely to shift radial-dorsally as a result of degeneration or rupture of the ligaments around the TMC joint. The TMC joint has a structure in which the distribution of subchondral bone density is predisposed to gather on the RD side of the trapezium during pinch and opposition^7,28^. Moreover, the structure of the trapezium, in which the articular surface has a concave shape in coronal view, is more likely to gather stress on the RD side of the articular surface of the trapezium if the first metacarpal shifts radial-dorsally. From these factors, we surmise that the distributions of subchondral bone density were higher in the RD region if the articular surface of the trapezium was more radially inclined (Fig. 4).

The stability of the TMC joint depends on muscular activity and ligament tension. In various studies, the anterior oblique ligament, the intermetacarpal ligament and the dorsoradial ligament have all been proposed as primary stabilizers of the TMC joint^3–5,25,29–34^. The anterior oblique ligament and dorsoradial ligament is considered to prevent translation of the first metacarpal to the dorsal side. Vincent et al. reported that laxity of the beak ligament induced radial translation of the first metacarpal, so the main contact area of the TMC joint shifted from the volar side to the dorsal side^26^. In terms of the etiology of TMC osteoarthritis, many investigators have theorized that ligamentous laxity of the TMC joint leads to an incongruous relationship between joint surfaces^28,29,35^. This incongruity is thought to lead to smaller contact areas and thus greater contact stresses in certain areas of the joint, leading to degeneration and osteoarthritis^7,28^. Matthew et al. suggested that there was significant cartilage wear on the RD quadrant of the trapezium in advanced-stage osteoarthritis^9^. Our results showed a larger high-density area in the RD region of trapeziums showing a higher TI. Thus, not just ligamentous laxity but also the bony morphology of the trapezium could be involved in TMC osteoarthritis. We surmise that there may be a correlation between the bony morphology of the trapeziums with a high TI and the progression of TMC osteoarthritis.

In previous reports relevant to the relationship between bony morphology of the TMC joint and TMC osteoarthritis, cases of advanced TMC osteoarthritis tilted more toward the radial side of the trapezium and the dorsal side of the first metacarpal^20,21^. From these reports, the bony morphology of the TMC joint is suspected to be involved in the commitment to TMC osteoarthritis. However, these reports did not prove whether the bony morphology causes TMC osteoarthritis or bony morphological alterations occur as a result of the progression of TMC osteoarthritis. Bettinger et al. suggested that the bony morphology of trapeziums that tilted more radially tended to cause TMC osteoarthritis, based on the anteroposterior view of the radiograph^20^. We also think that differences in bony morphology of the trapezium are a cause of TMC osteoarthritis because of our results showing that the distributions of subchondral bone density of the RD region on the articular surface of the trapezium were higher in cases having a higher TI. Miura et al. showed that average volar tilt in patients with TMC osteoarthritis increased significantly compared to those without TMC osteoarthritis and that dorsal translation of the first metacarpal was significantly higher in participants with TMC osteoarthritis^21^. However, our results showed that bony morphology of the first metacarpal was not involved in the distributions of subchondral bone density on the TMC joint surface. From these results, we surmise that the changes in the first metacarpal might occur as a result of progression of TMC osteoarthritis, and the bony morphology of the trapezium might be more involved in TMC osteoarthritis than that of the first metacarpal. Hence, the bony morphology of the trapezium is suggested to influence the distributions of subchondral bone density in the TMC joint and that trapeziums showing a more radial tilt have increased the distributions of subchondral bone density in the RD region of the trapezium, leading to TMC osteoarthritis. We believe that our results have the potential to elucidate osteoarthritic mechanisms of the TMC and that this characteristic morphology of the trapezium may provide a predictive factor for the occurrence of TMC osteoarthritis. However, since we did not examine patients with osteoarthritis, comparisons between normal and osteoarthritis patients need to be done.

The present study showed several limitations. First, the sample number was small. We therefore think that investigation of further samples is required. Second, stresses in the articular surfaces of the trapezium and first metacarpal were measured indirectly, and the current results are based on the assessment of bone mineral density in both articular surfaces. Third, the background characteristics of participants were not constant, so stresses on the TMC joint underwent changes caused by employment, life environment, history of sports, and so on. Fourth, the current study did not investigate the effect of ligamentous laxity. Fifth, there is no consensus about the definition of the high-density area. There are various definitions of this area in previous reports^13–19^. We selected the upper one-third of HU values as the high-density area according to previous reports^15,16^. The results might be different if we selected a different area for the HU values such as the upper-quarter. Finally, this study focused only on the coronal plane of the trapezium and sagittal plane of the first metacarpal. In the future, we need to clarify the detailed mechanisms of the TMC joint by investigating relationships between 3-dimensional bony morphology and the distributions of subchondral bone density.

In conclusion, the results derived from CT-OAM suggest that the distribution of subchondral bone density tends to be more concentrated in the RD region of the articular surface of trapeziums with more trapezial inclinations. For this reason, bony morphology of the trapezium may become an important factor in the diagnosis and treatment of TMC osteoarthritis. CT-OAM provides clinical information for analyzing loading conditions associated with the TMC joint. However, further study is required to clarify other pathological mechanisms involved in the relationship between bony morphology of the TMC joint surface and TMC osteoarthritis.

## Data Availability

The datasets generated during and/or analysed during the current study are available from the corresponding author on reasonable request. All data generated or analysed during this study are included in this published article.
